# NeuroFAIR curator: an AI-assisted framework for metadata enrichment, quality screening, curation prioritization, and FAIR readiness of brain tumor MRI data

**DOI:** 10.3389/fninf.2026.1907796

**Published:** 2026-09-16

**Authors:** Faizan Ullah, Zaheer Abbas, Mohmmad Abrar, Farhan Amin, N. Jon Shah

**Affiliations:** 1 Institute of Neuroscience and Medicine 4, INM-4, Forschungszentrum Jülich, Jülich, Germany; 2 Faculty of Computer Studies, Arab Open University, Muscat, Oman; 3 School of Computer Science and Engineering, Yeungnam University, Gyeongsan, Republic of Korea; 4 Department of Neurology, RWTH Aachen University, Aachen, Germany; 5 JARA-BRAIN – Translational Medicine, Aachen, Germany

**Keywords:** active curation, annotation integrity, anomaly detection, brain MRI, data curation, FAIR data, metadata enrichment, neuroinformatics

## Abstract

**Purpose:**

Public brain tumor MRI datasets are widely reused for classification and segmentation, but their metadata structure, technical annotation consistency, image quality, and FAIR readiness are seldom evaluated within a unified workflow. This study proposes NeuroFAIR Curator, an AI-assisted framework for metadata enrichment, computational quality screening, sample prioritization, and FAIR readiness assessment. The framework is intended for data stewardship and does not determine the clinical correctness of tumor annotations.

**Methods:**

NeuroFAIR Curator was evaluated on BRISC 2025, a two-dimensional contrast-enhanced T1-weighted MRI dataset distributed as JPEG images and PNG masks. A unified curation manifest combined images, masks, labels, split information, inferred anatomical planes, sequence descriptors, technical consistency checks, and quality indicators. A ResCNNClassifier generated label-confidence signals, while a UNet generated segmentation-derived uncertainty and agreement measures. Grad-CAM with deletion area under the curve (DAUC) supported classifier interpretation. Class- and plane-specific 1.5 × IQR limits were used for exploratory mask-area morphology screening. Seven normalized curation signals were fused into a Curation Uncertainty Index (CUI), and FAIR readiness was evaluated using an 18-sub-criterion rubric.

**Results:**

The framework generated 6,000 sample-level records linked with 15,586 external manifest entries and processed 4,793 image-mask pairs. Classification achieved 98.30% accuracy, 98.53% macro F1, and 99.79% macro AUC. Segmentation achieved Dice 82.30%, IoU 72.69%, precision 83.64%, recall 84.61%, Hausdorff distance 7.84 pixels, and HD95 4.11 pixels at a validation-selected threshold of 0.95. Exploratory morphology screening flagged 181 of 4,793 masks (3.78%) as statistically atypical; these flags were not interpreted as confirmed annotation errors. The mean Grad-CAM DAUC was 0.2996. CUI ranking captured 49 flagged samples at a 1% review budget. Findability improved from 87.50 to 100.00 and Interoperability from 36.00 to 91.98, while Accessibility and Reusability remained unchanged under the declared rubric.

**Conclusion:**

NeuroFAIR Curator converts a public brain tumor MRI corpus into a metadata-enriched, technically checked, quality-screened, review-prioritized, and FAIR-aligned neuroinformatics resource. Its morphology flags are exploratory computational signals for subsequent assessment rather than clinical judgments of mask correctness.

## 1 Introduction

The wider availability of public neuroimaging datasets has accelerated the development of artificial intelligence-based methods for brain disease analysis, neuro-oncology, and computational neuroscience. Open datasets support reproducible experiments, cross-study comparisons, and rapid benchmarking of classification and segmentation models. The value of a public neuroimaging resource, however, depends on more than the number of images it contains or the performance achieved by downstream models (Poldrack et al., 2013). It also relies on the quality of the metadata, consistency of the annotation, reliability of the file organization, clear provenance, and compliance with the F.A.I.R. (Findable, Accessible, Interoperable, Reusable) criteria for its scientific usefulness. Key to responsible stewardship of scientific data is the FAIR philosophy, which emphasizes that scientific data and its metadata should be understandable, searchable, machine-readable, and reusable across computational environments (Wilkinson et al., 2016). Traceable research workflows, structured data management, and transparent computational environments are essential for reproducible neuroimaging. BrainInsights provides a pipeline for neuroimaging preprocessing, analysis, and interpretation using conventional statistical and machine-learning methods (Selvakumar et al., 2026).

The need for well-annotated, standard, and validated metadata is especially high in neuroinformatics due to the fact that brain imaging datasets may contain many modalities, anatomical planes, preprocessing steps, imaging sequences, labels, masks, and task-specific annotations. Community standards such as the Brain Imaging Data Structure have improved the organization and description of neuroimaging data by providing a consistent format for files and metadata (Gorgolewski et al., 2016). Recent work has further emphasized that BIDS continues to evolve in response to the increasing scale and diversity of neuroscience datasets, including the need to support multimodal and longitudinal resources more effectively (Poldrack et al., 2024). Similarly, FAIR m-BIDS has been proposed to strengthen multimodal brain data utilization by aligning neuroimaging organization more closely with FAIR principles and improving interoperability across data entities (Mirhosseini et al., 2026). Together, these developments reflect a shift in neuroimaging research from simple data sharing to structured, validated, and reusable data ecosystems. Studies of laboratory and real-world neurophysiology data likewise indicate that scalable reuse requires standardized containers, structured annotations, and machine-readable descriptors, supporting the need for curation pipelines that convert heterogeneous files into interoperable research resources (Ganzetti et al., 2025; Shahrokhi et al., 2026).

Despite this progress, many public medical imaging datasets distributed through general repositories are still used directly for model training without a systematic evaluation of their curation state. Brain tumor MRI datasets are often used as ready-made inputs for classification or segmentation pipelines. This use often presumes that folder names encode the correct diagnostic labels, masks correspond to the correct images, anatomical plane tokens are accurate, and documentation reflects the actual dataset structure. While these assumptions are convenient for the development of a model, they can result in hidden problems if metadata are incomplete, if labels are inconsistent, if the masks are missing or abnormal, or if image quality is inconsistent from sample to sample. In recent biomedical image-analysis guidelines, task definition, the quality with which tasks are annotated, the selection of suitable metrics, and the characteristics of the data sets are all emphasized as important aspects for reliable evaluation of the models (Maier-Hein et al., 2024). Research on medical image segmentation based on heterogeneous/multi-source annotations also indicates that the uncertainty of the annotation can significantly impact the model learning and the interpretation of the results (Wang et al., 2025).

During the process of curating the datasets, AI can contribute to solving these issues. In addition to clinical labels or tumor segmentation, AI tools can analyze dataset structure, add additional metadata, identify anomalies, assess the integrity of annotations, and identify and prioritize samples that may be difficult to identify and prioritize samples with high curation uncertainty. In cases where large health data ecosystems include numerous data sources that are diverse in their storage, structure, and formats, one way to make these more usable is by leveraging AI-based data curation (de Zegher et al., 2024). There have been recent efforts to generate and standardize biomedical metadata with retrieval-augmented language models, ontological priors, and AI-generated templates, which have demonstrated potential to enhance the completeness and consistency of biomedical metadata (Tinn et al., 2025; Sundaram et al., 2026). The research shows that data stewardship is a prerequisite to downstream predictive modeling for scalable AI systems in Neuroinformatics. The BRISC 2025 Brain Tumor MRI Dataset is a valuable case study. This dataset includes 6,000 T1-weighted MRI images with contrast enhancement across tumor types (glioma, meningioma, pituitary tumor, and no tumor), split into predefined training and testing sets, pixel-level segmentation masks, and three anatomical views (axial, coronal, and sagittal) for each image (Fateh et al., 2025). BRISC was introduced to aid brain tumor segmentation and classification; it is useful as a benchmark dataset. Its form also poses a higher neuroinformatics question: How can it be converted into a resource for reliable reuse that is public, re-usable, FAIR, and full of metadata and annotation, and prioritized for review? This question is distinct from conventional classification or segmentation objectives because it focuses on the quality and reusability of the dataset itself rather than only on the accuracy of a diagnostic model.

To address this gap, this study proposes NeuroFAIR Curator, an AI-assisted framework for metadata enrichment, technical annotation screening, exploratory mask morphology characterization, image-quality analysis, uncertainty-driven curation prioritization, and FAIR readiness evaluation. The framework does not determine whether a statistically unusual tumor mask is anatomically incorrect. Instead, it identifies objective technical inconsistencies and statistically atypical characteristics that may support subsequent expert-led curation.

The main contributions of this work are as follows:

- NeuroFAIR Curator integrates metadata enrichment, classification-derived label screening, segmentation-derived uncertainty, objective technical annotation checks, exploratory mask morphology screening, image-quality analysis, CUI-based prioritization, and FAIR readiness evaluation.
- A transparent seven-component Curation Uncertainty Index combines classification uncertainty, segmentation uncertainty, statistical/structural mask evidence, metadata incompleteness, image-quality anomaly, image-mask mismatch, and metadata-validation error.
- A unified sample-level curation manifest links 6,000 framework records with 15,586 external manifest entries and carries inferred descriptors, technical checks, quality flags, model-derived signals, and FAIR descriptors.
- FAIR readiness, active-curation capture, threshold selection, and CUI sensitivity are quantified using explicit and reproducible procedures

The paper next reviews related work on FAIR neuroinformatics and AI-assisted curation, then describes the NeuroFAIR Curator framework and methodology. The experimental results are followed by the discussion, limitations, future directions, and conclusion.

## 2 Related work

### 2.1 FAIR Neuroinformatics and scalable brain data stewardship

With the increasing volume of neuroscience data, there is a growing need for structured, machine-readable, and reusable data management practices. FAIR neuroinformatics is not just about data sharing, but about ensuring that metadata is complete and has a provenance history, is semantically consistent, has been validated through workflow, and has infrastructure to support reuse on a large scale. Martone (2024) pointed out that the landscape of data sharing in neuroscience has moved beyond the deposition of resources in individual repositories to a distributed ecosystem of specialized resources, and FAIR implementation is crucial, as neuroscience data exists across a variety of modalities, spatial scales, temporal resolutions, and experimental designs. Similarly, Reer et al. (2023) noted the need for careful implementation of FAIR principles in human neuroscience data, as legal, ethical, and technical issues affect the ability to access, interpret, and reuse datasets for AI research.

New research also suggests that, without improvements to the data, such as making them more interlinked, consistent and properly annotated, FAIR policies will not help much. Poveda et al. (2026) pointed out that FAIR data policy can be turned into a compliance task if it is not accompanied by infrastructure that can systematically capture, curate, and link research data. This is a particular concern for public neuroimaging data sets, as data availability does not necessarily entail full or consistent metadata, labels, masks, and documentation. Similarly, the Frontiers Research Topic on AI-assisted data management and curation in neuroinformatics presents AI-assisted metadata enrichment, schema validation, ontology integration, and active learning-based annotation as key challenges for the large-scale reusability of neuroscience data (Dar et al., 2024). All these studies suggest practical frameworks for the curation of the datasets in the context of neuroinformatics and the assessment and enhancement of their readiness for downstream modeling.

### 2.2 Metadata enrichment and AI-assisted data curation

Metadata enrichment is important for FAIR data reuse as it adds context to make datasets searchable, interpretable, interoperable, and reusable. Traditional metadata curation typically relies on manual input, fixed templates, and repository-specific metadata needs, which can be challenging to scale for thousands of files, disparate file structures and documentation, or incomplete metadata. AI-based curation approaches have explored the application of machine learning, natural language processing (NLP), and large language models (LLMs) to enable metadata completion, standardize terminology, and support quality assurance. Jariwala et al. (2025) demonstrated that curating metadata with the help of AI can enhance the accuracy and interoperability of scientific repositories by incorporating automated metadata suggestions into the curation workflow. Other aspects of the metadata ecosystem and AI also suggest that FAIR and AI-ready data need metadata workflows that make use of structured metadata standards, domain vocabularies, and automated quality control (Greenberg et al., 2026).

AI-driven curation can enhance the consistency and usefulness of complex health data sources in biomedical data management. This is particularly helpful if metadata fields are missing, or if there are inconsistencies in naming conventions or a lack of documentation describing the file’s actual structure. But the metadata produced using AI is not necessarily accurate. NeuroFAIR Curator takes this approach: metadata enrichment is not just a preprocessing step, but part of a full curation pipeline that involves parsing file names, extracting plane-aware metadata, validating image and mask quality, encoding quality-control indicators, and scoring for FAIR readiness.

### 2.3 Brain imaging standards, medical imaging frameworks, and reproducibility

Standardized data organization is essential for reproducible neuroimaging research and provides a consistent foundation for representing imaging files and associated metadata across studies. This is an important context for organizing imaging files and imaging data information across studies and a foundation provided by the Brain Imaging Data Structure and its recent extensions. Recent efforts in FAIR mBIDS have shown the need for a more robust entity-level metadata representation for multimodal brain data use, particularly in the case of the same entity or data being represented in multiple modalities or studies (Mirhosseini et al., 2026). If the brain tumor MRI datasets are to be used for more than one modeling experiment, then classification labels, segmentation masks, anatomical planes, imaging sequences, and quality indicators derived from the MRI must be consistent with each other.

The reproducibility of medical imaging AI also relies on the use of open-source frameworks that have been developed to standardize preprocessing, model development, annotation, and model evaluation. MONAI is an open-source, domain-specific framework that supports reproducible deep-learning workflows for medical-image classification, segmentation, registration, and deployment (Cardoso et al., 2022). MONAI Label is a previously developed framework for AI-assisted interactive annotation, in which model predictions and expert corrections are iteratively incorporated to refine image labels and segmentation masks (Díaz-Pinto et al., 2024) These frameworks show that dependable medical imaging AI not only relies on model architectures, but also on constant information loading, annotation management, transformation pipelines, and validation tools. NeuroFAIR Curator takes this forward as a curation layer for a public brain MRI set, not just a predictive model.

### 2.4 Annotation integrity and uncertainty in medical image segmentation

Medical image annotations are often treated as reference standard, but they are affected by inter-observer variability, image quality, task ambiguity, and the difficulty of defining precise lesion boundaries. In segmentation, uncertainty can arise both at the pixel level and at the image-level. Li et al. (2025) evaluated uncertainty quantification in medical image segmentation and showed that multiple expert annotations can influence model uncertainty and performance interpretation. Such findings are important for dataset curation because disagreement, ambiguity, or abnormal segmentation behavior should not be ignored when a dataset is reused for benchmarking. Recent studies have highlighted the significance of uncertainty in medical image segmentation, which can arise from model uncertainty and from disagreements between expert annotators. The importance of explicitly modeling inter-rater variability was highlighted by the QUBIQ benchmark, and the example of expert disagreement was shown to be useful for calibrated, single-pass uncertainty estimation (Abutalip et al., 2024; Li et al., 2024). In addition to being evaluated visually, visual explanations should be evaluated quantitatively, as deletion and insertion AUC measure its fidelity: while they should be interpreted together with localization or calibration measures (Gomez et al., 2022), they are complementary. For manual delineation of postoperative glioblastomas from MRI, large variations in inter- and intra-observer measurements have been reported, suggesting that model–reference disagreement is not necessarily a sign of annotation errors (Helland et al., 2023).

Recent studies have also moved toward uncertainty-aware quality-control rather than relying only on global segmentation metrics. Image-specific segmentation quality assessment has been proposed to account for the fact that different input images can have different levels of difficulty, making a single global threshold insufficient for accepting or rejecting predicted masks (Fournel et al., 2025). Similarly, uncertainty-guided annotation frameworks have shown that model uncertainty can support human-in-the-loop correction by identifying regions where clinician review is most needed (Khalili et al., 2024). These studies support the idea that uncertainty should be used not only to interpret model predictions, but also to guide dataset review and annotation refinement. In the present study, this principle is incorporated through the Curation Uncertainty Index, which combines classification uncertainty, segmentation uncertainty, mask abnormality, metadata incompleteness, anomaly scores, and validation flags to rank samples for review.

### 2.5 CUI guided active curation and review prioritization

Active learning has become an important strategy for reducing annotation cost in medical image analysis. Instead of requiring experts to review or annotate every sample, active learning methods attempt to identify the most informative, uncertain, diverse, or potentially erroneous samples for human attention. Recently, medical image-segmentation research has focused on uncertainty-reinforced sampling, cold-start learning, and selective review methods that promote more efficient image annotations without compromising model accuracy (Zhu et al., 2025). This is relevant for neuroinformatics curation since the experts’ time is limited and they have to work with thousands of image and mask pairs.

Human-in-the-loop (HWIL) systems also understand the value of the expert in the loop—not in the automation. In fact, AI can serve as a tool to focus on reviewing, provide candidate corrections, and either uncover uncertainty while remaining under human supervision, or leave the final interpretation to the human radiologist (Díaz-Pinto et al., 2024; Khalili et al., 2024; Zhu et al., 2025). NeuroFAIR Curator is an implementation of this principle for dataset curation. The framework does not assume that uncertain labels or masks are incorrect. Instead, it produces validation flags and CUI-ranked samples that enable the curators to narrow down to those with the highest relative risk. This is important because some ambiguously collected samples in public datasets may need to be reviewed by experts instead of automatically deleted. BRISC 2025 is a newly released brain tumor MRI dataset, consisting of 6,000 contrast-enhanced T1-weighted MR images in glioma, meningioma, pituitary tumor, and no-tumor categories, with anatomical images and segmentation masks (Fateh et al., 2026).

Current methods typically focus on metadata standardization, support for metadata annotations, uncertainty estimation, and FAIR assessment separately. These functions are combined in a sample-level workflow for NeuroFAIR Curator that generates a combined manifest, technical-screening and exploratory-morphology signals, Curation Uncertainty Index, and pre- and post-curation FAIR readiness scores.

## 3 Materials and methods

### 3.1 Study design and overall workflow

NeuroFAIR Curator (NFC) is an AI-driven neuroinformatics curation system to enhance the organization, metadata richness, technical annotation consistency, quality transparency, and FAIR readiness of brain tumor MRI datasets. It was not designed as a diagnostic prediction tool, but as a curation workflow to transform a research-grade MRI corpus into a machine-actionable, structured resource for the community to reuse. The flowchart seen in Figure 1 outlines the process of ingestion of raw MRI data, workflows, and reporting to FAIR readiness. All intermediate outputs are kept along the way, including metadata fields, model predictions, quality and annotation flags, CUI scores, active-curation rankings, and FAIR dimension scores.

**Figure 1:**
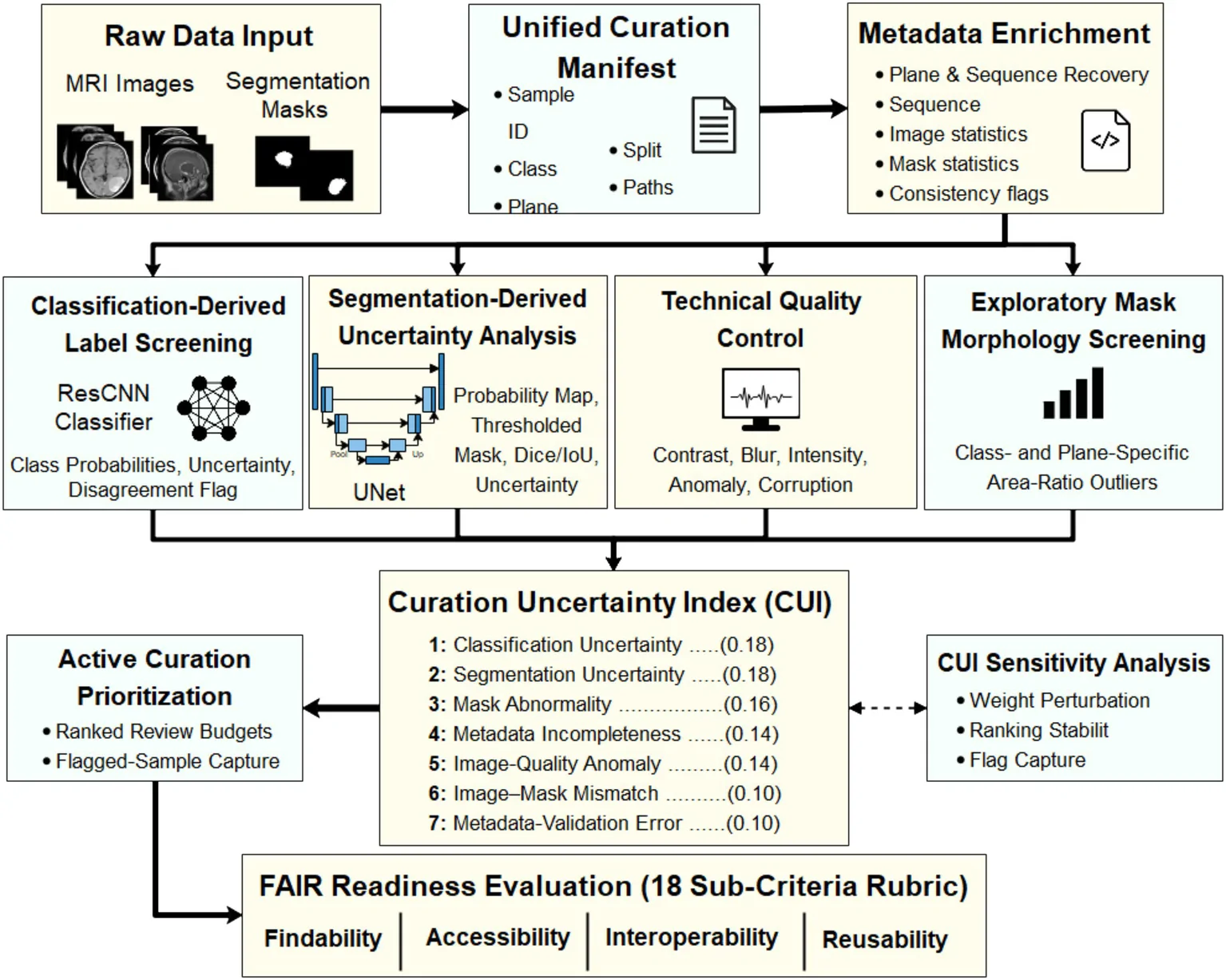
Conceptual architecture of NeuroFAIR Curator. Raw MRI images, masks, and external records are combined in a unified manifest. The pipeline performs metadata enrichment, classification-derived label screening, segmentation-derived uncertainty analysis, technical quality control, exploratory mask morphology screening, CUI computation and sensitivity analysis, active curation prioritization, and FAIR readiness evaluation. MRI images displayed in the figures originate from the BRISC 2025 dataset. Source: BRISC: Annotated Dataset for Brain Tumor Segmentation and Classification. Creative Commons Attribution 4.0 International (CC BY 4.0); the dataset is cited in the manuscript.

### 3.2 Proposed framework

NeuroFAIR Curator is designed to be an auditable process comprising four stages: building the manifest, enriching the metadata, screening labels using classification-derived confidence and characterizing masks using segmentation-derived agreement, checking the labels based on rules, analyzing the image quality, calculating CUIs, ranking the curation activities, and assessing the readiness to be FAIR. The per-sample processing and ranking procedure is summarized in Algorithm 1.

### 3.3 Dataset description

The framework was used on the BRISC 2025 brain tumor MRI dataset (Fateh et al., 2026) consisting of classification samples and segmentation image-mask pairs. There are four target classes: glioma, meningioma, pituitary, and no-tumor. The MRI images cover axial, coronal, and sagittal anatomical planes. The classification subset contains 6,000 images, while the segmentation subset contains 4,793 image-mask pairs. The classification data are divided into 5,000 training samples and 1,000 test samples. The dominant native image size is 512×512 , and the framework generates 6,000 sample-level records linked with the 15,586 external manifest entries, yielding 21,586 unified curation records. BRISC consists of two-dimensional contrast-enhanced T1-weighted MRI images stored in JPEG format, with corresponding binary segmentation masks stored in PNG format. The images represent axial, coronal, and sagittal views and do not constitute spatially registered three-dimensional volumes. The manifest construction stage converts heterogeneous file-level information into a structured sample-level representation. Each record is assigned a unique sample identifier and linked with available paths, labels, split information, inferred plane, inferred sequence, image dimensions, mask availability, and filename consistency indicators. The manifest therefore serves as the central data structure for all subsequent curation operations.

For each sample i , the initial metadata vector is defined as:

$$z_{i}^{0}=[id_{i},p_{i}^{\mathrm{img}},p_{i}^{\text{mask}},y_{i},s_{i},r_{i}]$$ (1)

where $id_{i}$ is the sample identifier, $p_{i}^{\mathrm{img}}$ is the image path, $p_{i}^{\text{mask}}$ is the mask path when available, $y_{i}$ is the recorded class label, $s_{i}$ is the split assignment, and $r_{i}$ represents the raw filename or folder-level descriptors.

### 3.4 Metadata enrichment strategy

Metadata enrichment is performed to convert the raw dataset structure into a richer curation manifest. The enriched metadata vector combines original descriptors, inferred fields, computed image statistics, mask statistics, annotation consistency flags, quality indicators, model-derived signals, and FAIR descriptors. The final enriched metadata vector is defined as:

$$z_{i}=[z_{i}^{\text{orig}},z_{i}^{\inf},z_{i}^{\mathrm{img}},z_{i}^{\text{mask}},z_{i}^{\mathrm{ann}},z_{i}^{\mathrm{qc}},z_{i}^{\text{model}},z_{i}^{\text{FAIR}}]$$ (2)

where $z_{i}^{\text{orig}}$ contains original metadata, $z_{i}^{\inf}$ contains inferred descriptors such as anatomical plane and sequence, $z_{i}^{\mathrm{img}}$ contains image statistics, $z_{i}^{\text{mask}}$ contains mask statistics, $z_{i}^{\mathrm{ann}}$ contains annotation consistency indicators, $z_{i}^{\mathrm{qc}}$ contains quality-control signals, $z_{i}^{\text{model}}$ contains classification and segmentation outputs, and $z_{i}^{\text{FAIR}}$ contains FAIR-related descriptors.

For each image $x_{i}$ , the framework computes intensity and quality descriptors:

$$\mu_{i}=\frac{1}{|\Omega |}\sum_{p\in \Omega }x_{i}(p)$$ (3)

$$\sigma_{i}=\sqrt{\frac{1}{|\Omega |}\sum_{p\in \Omega }{\left(x_{i}(p)-\mu_{i}\right)}^{2}}$$ (4)

where Ω is the image domain, $\mu_{i}$ is the mean intensity, and $\sigma_{i}$ is the intensity standard deviation. Additional descriptors include contrast, entropy, blur score, and corruption status. When a segmentation mask is available, the mask area ratio is calculated as:

$$\rho_{i}=\frac{|m_{i}|}{|\Omega |}$$ (5)

where $|m_{i}|$ is the number of foreground pixels in the provided mask. This value is used to identify unusually small or large masks relative to the class and plane distribution. Across all quality and annotation flags, 456 unique samples were flagged with at least one issue, 184 samples (3.07%) carried multiple flags simultaneously, and the mean number of flags per flagged sample was 1.49. Of the 6,000 total samples, 5,544 had zero flags.

### 3.5 Classification based label validation

The classification component is used as a label-validation instrument, and the proposed ResCNNClassifier architecture is retained unchanged. Images are resized to 128 × 128, standardized per image, and subjected to mild MRI-safe augmentation comprising horizontal reflection, small in-plane rotation, mild zoom, and conservative brightness and contrast perturbations. Training uses effective-number class weights (*β* = 0.999), weighted cross-entropy with label smoothing of 0.02, AdamW with an initial learning rate of 1.2 × 10^−3^ and weight decay of 3 × 10^−4^, and MixUp with *α* = 0.20 applied with probability 0.35. A five-epoch linear warm-up is followed by cosine learning-rate decay, and an exponential moving average of the model weights is maintained with decay 0.9993. Raw and EMA checkpoints are compared using validation macro F1 and validation loss; the base checkpoint is selected. No TTA, horizontal-flip TTA, and flip-plus-zoom TTA are compared using validation data only, and no TTA is selected. The held-out test set is evaluated only after the checkpoint and inference policy are fixed. The model maps each image to a probability vector over classes:

$$p_{i}=f_{\mathrm{cls}}(x_{i};\theta_{\mathrm{cls}})\in {\left[0,1\right]}^{C}$$ (6)

where $f_{\mathrm{cls}}$ is the classification model, $\theta_{\mathrm{cls}}$ denotes its trainable parameters, and C=4 corresponds to glioma, meningioma, pituitary, and no-tumor.

The predicted class is obtained as:

$${\hat{y}}_{i}=\arg\max_{c\in C}p_{i}(c)$$ (7)

Classification confidence is defined as:

$$\mathrm{con}f_{i}^{\mathrm{cls}}=\max_{c\in C}p_{i}(c)$$ (8)

and classification uncertainty is defined as:

$$u_{i}^{\mathrm{cls}}=1-\mathrm{con}f_{i}^{\mathrm{cls}}$$ (9)

To assess label consistency, the recorded label $y_{i}$ is compared with the model prediction ${\hat{y}}_{i}$ . A label disagreement indicator is defined as:

$$d_{i}^{\mathrm{cls}}=\{\begin{array}{cc} 1, & {\hat{y}}_{i}\neq y_{i}\\ 0, & {\hat{y}}_{i}=y_{i} \end{array}$$ (10)

The disagreement indicator and classification uncertainty are stored in the enriched manifest as curation signals rather than as diagnostic outputs.

### 3.6 Segmentation-derived mask screening

The segmentation component is used as a computational screening instrument rather than as a clinical validator of the supplied masks. A UNet implemented in PyTorch is trained on the segmentation image-mask pairs. For each image, the $x_{i}$ , model produces a probability map:

$${\hat{m}}_{i}^{\text{prob}}=f_{\mathrm{seg}}(x_{i};\theta_{\mathrm{seg}})$$ (11)

where $f_{\mathrm{seg}}$ is the segmentation model and $\theta_{\mathrm{seg}}$ denotes its trainable parameters. A binary predicted mask is generated using the tuned threshold τ :

$${\hat{m}}_{i}=\{\begin{array}{cc} 1, & {\hat{m}}_{i}^{\text{prob}}\geq \tau \\ 0, & {\hat{m}}_{i}^{\text{prob}}<\tau \end{array}$$ (12)

A binary predicted mask is generated by applying an operating threshold to the probability map. Screening is summarized τ=0.95 using Dice score, Intersection over Union, precision, recall, Hausdorff distance, HD95.boundary quality, and segmentation uncertainty. These measures quantify model-reference agreement and are used as curation signals; they do not establish clinical correctness. Dice score is defined as:

$$\mathrm{Dic}e_{i}=\frac{2|m_{i}\cap {\hat{m}}_{i}|}{|m_{i}|+|{\hat{m}}_{i}|+\in }$$ (13)

and IoU is defined as:

$$\mathrm{Io}U_{i}=\frac{|m_{i}\cap {\hat{m}}_{i}|}{|m_{i}\cup {\hat{m}}_{i}|+\in }$$ (14)

where ∈ is a small constant used to avoid division by zero. Segmentation uncertainty is computed as:

$$u_{i}^{\mathrm{seg}}=1-\mathrm{Dic}e_{i}$$ (15)

Metrics were computed for each two-dimensional image-mask pair and averaged over the held-out test set. When a reference mask contained multiple isolated components, the complete binary mask was evaluated as a single slice-level reference. Hausdorff distance and HD95 were computed on the 160 × 160 evaluation grid and are reported in pixels because reliable pixel-spacing metadata were unavailable. Candidate thresholds from 0.05 to 0.95 in increments of 0.05 were evaluated only on the validation partition. The selected threshold was τ* = argmaxτ Diceval(*τ*). This procedure selected τ* = 0.95, with a maximum validation Dice of 0.8108, and the threshold was fixed before test evaluation.

As shown in Figure 2, the segmentation operating threshold was selected exclusively from the validation set by evaluating candidate probability thresholds from 0.05 to 0.95. Validation Dice increased progressively across the tested range, reaching its maximum value of 0.8108 at *τ* = 0.95. Accordingly, 0.95 was selected as the final operating threshold and fixed before evaluation on the held-out test set, thereby preventing test-set information from influencing threshold selection.

**Figure 2:**
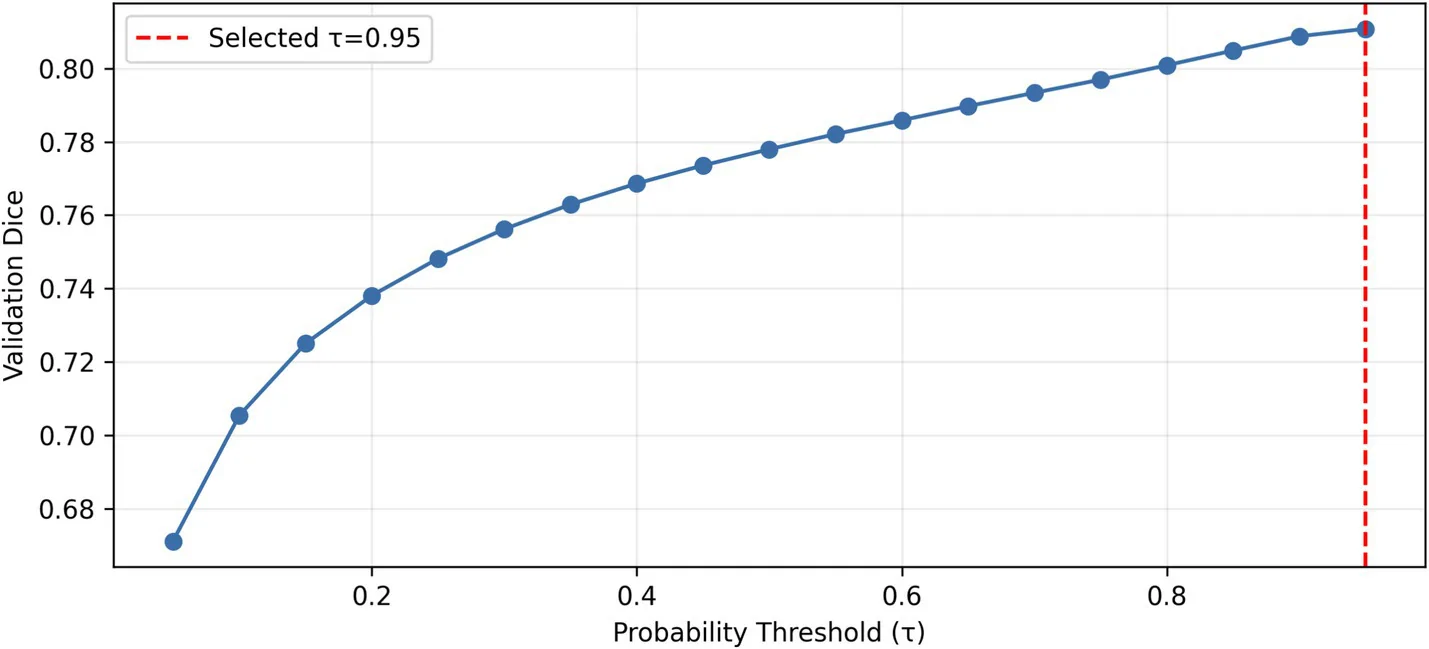
Validation Dice is shown for thresholds from 0.05 to 0.95; 0.95 achieved the maximum validation Dice of 0.8108 and was fixed before test evaluation.

### 3.7 Explainability analysis (grad-CAM and DAUC)

To make the classification part more interpretable, Grad-CAM (Gradient-weighted Class Activation Mapping) saliency maps are computed from the last convolutional layer of the ResCNNClassifier for the top 20 images in the test set that were most ambiguous. Saliency maps show which parts of the image are most important for the classification. For these maps, the value of the map is quantitatively assessed by the Deletion Area Under Curve (DAUC): the map is progressively masked starting from the most salient information to the least salient, and each time the resulting class probability is recorded. The DAUC is calculated using the trapezoidal rule. Localization fidelity is associated with a lower DAUC.

### 3.8 Technical annotation integrity and exploratory morphology screening

Technical annotation consistency was assessed using objective file- and manifest-level checks: missing masks for tumor samples, unexpectedly large masks for no-tumor samples, empty tumor masks, image-mask dimension mismatch, filename-label mismatch, filename-split mismatch, filename-mask mismatch, unknown anatomical plane, and corruption status. These checks identify directly observable technical inconsistencies.

For each sample, an annotation inconsistency score is defined as:

$$a_{i}=\sum_{k=1}^{K}\alpha_{k}I_{\mathrm{ik}}^{\mathrm{ann}}$$ (16)

The objective annotation indicators include missing tumor mask, large no-tumor mask, empty tumor mask, dimension mismatch, filename-label mismatch, filename-split mismatch, filename-mask mismatch, and unknown plane. Mask-area rarity was analyzed separately as an exploratory morphology signal and was not treated as evidence of annotation error. For exploratory morphology screening, supplied mask-area ratios were stratified by tumor class and anatomical plane. Within each class-plane stratum, the lower and upper limits were defined as Q1–1.5 × IQR and Q3 + 1.5 × IQR, respectively. A mask was flagged when its area ratio fell outside these limits:

$$\rho_{i}\langle L_{c,q}\text{or}\rho_{i}\rangle U_{c,q}$$ (17)

Where Q1 and Q3 are the first and third quartiles and IQR = Q3 − Q1 for $L_{c,q}$ and $U_{c,q}$ the corresponding class-plane distribution. The resulting flag represents statistical rarity only and does not establish anatomical incorrectness, clinical implausibility, or a requirement for automatic exclusion.

### 3.9 Image quality control and anomaly detection

Image quality control detects samples that may reduce dataset reuse quality. The framework evaluates low contrast, blur outliers, intensity outliers, anomaly flags, and corrupted samples. These indicators are calculated from image-level statistics and stored in the enriched manifest.

The image quality anomaly score is formulated as:

$$q_{i}=\beta_{1}I_{i}^{\text{contrast}}+\beta_{2}I_{i}^{\text{blur}}+\beta_{3}I_{i}^{\text{intensity}}+\beta_{4}I_{i}^{\text{anomaly}}+\beta_{5}I_{i}^{\text{corrupt}}$$ (18)

where $I_{i}^{\text{contrast}}$ , $I_{i}^{\text{blur}}$ , $I_{i}^{\text{intensity}}$ , $I_{i}^{\text{anomaly}}$ , and $I_{i}^{\text{corrupt}}$ are binary indicators for low contrast, blur outlier, intensity outlier, anomaly flag, and corruption status, respectively. The coefficients $\beta_{1},\ldots ,\beta_{5}$ control the relative contribution of each quality signal.

The corrupted sample indicator is defined as:

$$I_{i}^{\text{corrupt}}=\{\begin{array}{cc} 1, & x_{i}\;\text{cannot}\;\mathrm{be}\;\text{loaded or parsed}\\ 0, & \text{otherwise} \end{array}$$ (19)

This quality-control layer provides a transparent record of image-level conditions that may affect downstream reuse. Image contrast was defined as the difference between the 95th and 5th grayscale-intensity percentiles:

$$C_{i}=P_{95}(I_{i})-P_{5}(I_{i})$$ (20)

Images with $C_{i}<69.000$ , corresponding to the first percentile of the corpus distribution, were classified as low contrast. Blur was quantified using the variance of the Laplacian:

$$B_{i}=\mathrm{Var}(\nabla^{2}I_{i})$$ (21)

Images with $B_{i}<18.1565$ , corresponding to the first percentile, were classified as blur outliers. An intensity outlier was defined as an image satisfying at least one of the following conditions:

$$\mu_{i}\langle 20.5380,\mu_{i}\rangle 90.1127,\sigma_{i}\langle 27.7959,\sigma_{i}\rangle 77.6965$$ (22)

These limits were based on the distribution of means and standard deviations of the images and spanned the 1st and 99th percentile values. Global anomaly detection used an Isolation Forest with 150 trees, a contamination rate of 0.03 and random seed 42. Its input features included image mean, standard deviation, contrast, entropy, blur score, mask-area ratio, metadata incompleteness, mask abnormality and metadata-validation error.

### 3.10 Curation uncertainty index

The Curation Uncertainty Index provides a normalized sample-level score for computational review prioritization. Seven complementary signals were included: classification uncertainty, segmentation uncertainty, mask abnormality, metadata incompleteness, image-quality anomaly, image–mask mismatch, and metadata-validation error. For sample i , the CUI was defined as

$$\begin{array}{l} {\mathrm{CUI}}_{i}=0.18U_{i}^{c}+0.18U_{i}^{s}+0.16A_{i}^{m}+0.14M_{i}\\ +0.14Q_{i}+0.10D_{i}+0.10E_{i} \end{array}$$ (23)

where $U_{i}^{c}$ is classification uncertainty, $U_{i}^{s}$ is segmentation uncertainty, $A_{i}^{m}$ is the mask-abnormality score, $M_{i}$ is metadata incompleteness, $Q_{i}$ is the image-quality anomaly score, $D_{i}$ is the image–mask mismatch indicator, and $E_{i}$ is the metadata-validation error score. All components were restricted to [0,1] , and the component weights summed to 1.0.

Classification uncertainty was calculated from the maximum predicted class probability:

$$U_{i}^{c}=1-\max_{k}p_{\mathrm{ik}}$$ (24)

where $p_{\mathrm{ik}}$ is the predicted probability of class k . Segmentation uncertainty was calculated as the mean pixel-wise binary entropy of the predicted probability map:

$$U_{i}^{s}=\frac{1}{|\Omega_{i}|}\sum_{x\in \Omega_{i}}[\begin{array}{l} -p_{i}(x)\log_{2}p_{i}(x)-(1-p_{i}(x))\\ \log_{2}(1-p_{i}(x)) \end{array}]$$ (25)

Because binary entropy has a maximum value of 1 when the logarithm has base 2, this component was inherently bounded to [0,1] .

The mask-abnormality score combines objective technical mask indicators with the exploratory class- and plane-specific morphology flag. Because morphology rarity does not establish clinical error, this component is interpreted only as a curation-priority signal:

$$A_{i}^{m}=\frac{1}{4}\sum_{r=1}^{4}a_{\mathrm{ir}}$$ (26)

Metadata incompleteness was calculated as the fraction of missing values among ten required fields:

$$M_{i}=\frac{n_{i}^{\text{missing}}}{10}$$ (27)

where the required fields were sample identifier, image path, class label, split, anatomical plane, sequence, image format, image size, mask availability, and tumor/no-tumor status.

The image-quality anomaly score was obtained from an Isolation Forest fitted to image intensity, contrast, entropy, blur, mask-area, and metadata-quality descriptors. The negative Isolation Forest score was min–max normalized:

$$Q_{i}=\frac{s_{i}-\min_{j}(s_{j})}{\max_{j}(s_{j})-\min_{j}(s_{j})}$$ (28)

The image–mask mismatch term was binary:

$$D_{i}=I(\text{dimension mismatchfilename}–\text{mask mismatch})$$ (29)

The metadata-validation error score was the mean of five binary indicators covering dimension mismatch, filename–label mismatch, filename–split mismatch, filename–mask mismatch, and unknown anatomical plane:

$$E_{i}=\frac{1}{5}\sum_{r=1}^{5}e_{\mathrm{ir}}$$ (30)

The baseline weights were defined as a prespecified hierarchical prior and were not fitted using the held-out test set. The model-uncertainty tier received 0.36 and was divided equally between classification and segmentation uncertainty (0.18 each). Statistical and structural mask evidence received 0.16. Reuse-quality evidence received 0.28 and was divided equally between metadata incompleteness and image-quality anomaly (0.14 each). Sparse technical validation signals received 0.20 and were divided equally between image-mask mismatch and metadata-validation error (0.10 each). The complete weight vector therefore sums to one, and no single sparse binary flag can dominate the continuous uncertainty signals.

$$\begin{array}{l} {\text{Category}}_{i}\\ =\{\begin{array}{cc} \text{Clean sample}, & {\mathrm{CUI}}_{i}<0.20,\\ \text{Minor metadata issue}, & 0.20\leq {\mathrm{CUI}}_{i}<0.40,\\ \text{Annotation review required}, & 0.40\leq {\mathrm{CUI}}_{i}<0.65,\\ \text{High}-\text{risk sample}, & {\mathrm{CUI}}_{i}\geq 0.65. \end{array} \end{array}$$ (31)

A one-at-a-time sensitivity analysis varied each baseline component weight by −50%, −25%, +25%, and +50%. After each perturbation, all seven weights were renormalized to sum to one. CUI scores and rankings were recomputed, and robustness was quantified using flagged-sample capture at 1, 5, and 10% review budgets, Spearman correlation with the baseline ranking, and top-k overlap with baseline review sets.

### 3.11 Active curation prioritization

Active curation prioritization ranks samples according to their CUI values. The goal is to identify a small subset of samples that contains a higher concentration of curation flags than would be expected under random inspection.

Let B denote a review budget expressed as a percentage of the dataset. The number of selected samples is:

$$n_{B}=\frac{B}{100}N$$ (32)

The samples are sorted in descending order of CUI:

$$\mathrm{CU}I_{\left(1\right)}\geq \mathrm{CU}I_{\left(2\right)}\geq \cdots \geq \mathrm{CU}I_{\left(N\right)}$$ (33)

and the top $n_{B}$ samples are selected:

$$S_{B}=\{i_{\left(1\right)},i_{\left(2\right)},\ldots ,i_{\left(n_{B}\right)}\}$$ (34)

The flagged sample capture rate is calculated as:

$$\text{Capture}(B)=\frac{|S_{B}\cap F|}{|F|}$$ (35)

where F is the set of all flagged samples in the corpus.

This procedure provides a quantitative estimate of how effectively CUI ranking concentrates curation-relevant samples under different inspection budgets.

### 3.12 FAIR readiness evaluation

FAIR readiness is assessed before and after applying NeuroFAIR Curator. The evaluation is organized around the four FAIR dimensions: Findability, Accessibility, Interoperability, and Reusability. Each dimension is scored on a 0–100 scale based on the availability of structured metadata, standardized paths, recoverable descriptors, consistency checks, quality reports, technical annotation consistency indicators, and provenance-related fields.

The FAIR readiness vector is defined as:

R=[F,,,A,,,I,,,R] (36)

where F denotes Findability, A denotes Accessibility, I denotes Interoperability, and R denotes Reusability. The pre-curation and post-curation FAIR vectors are defined as:

$$R^{\mathrm{pre}}=[F^{\mathrm{pre}},,,A^{\mathrm{pre}},,,I^{\mathrm{pre}},,,R^{\mathrm{pre}}]$$ (37)

$$R^{\text{post}}=[F^{\text{post}},,,A^{\text{post}},,,I^{\text{post}},,,R^{\text{post}}]$$ (38)

The improvement vector is computed as:

$$\mathrm{\Delta R}=R^{\text{post}}-R^{\mathrm{pre}}$$ (39)

or equivalently:

$$\mathrm{\Delta R}=[F^{\text{post}}-F^{\mathrm{pre}},A^{\text{post}}-A^{\mathrm{pre}},I^{\text{post}}-I^{\mathrm{pre}},R^{\text{post}}-R^{\mathrm{pre}}]$$ (40)

This enables direct comparison of FAIR readiness before and after the curation workflow shown in Algorithm 1. The mathematical formulation of the NeuroFAIR Curator framework is provided in Equations 1– 40.

### 3.13 Evaluation metrics

Classification performance is evaluated using accuracy, macro precision, macro recall, macro F1 score, macro one-vs-rest AUC, and class-wise precision, recall, F1 score, and support. The classification metrics are computed on the held-out test set. Segmentation performance is evaluated using Dice score, IoU, precision, recall, Hausdorff distance and HD95, boundary quality, and validation Dice at the selected threshold. These metrics support computational mask screening rather than clinical annotation validation. Curation performance is evaluated using objective technical checks, exploratory morphologyflags, image quality flags, anomaly flagged samples, corrupted samples, CUI categories, active curation capture rate, and review reduction. FAIR readiness is evaluated using before, after, and improvement scores for each FAIR dimension.

### 3.14 Experimental setting

All experiments were performed with Python 3.12.13, PyTorch 2.10.0, CUDA 12.8, and random seed 42 in a CUDA environment with two NVIDIA Tesla T4 GPUs available, each with 15.64 GB of memory. Classification images were resized to 128 × 128. The unchanged ResCNNClassifier was trained for 120 epochs using AdamW, effective-number class weighting, weighted cross-entropy, label smoothing of 0.02, MixUp (*α* = 0.20; probability 0.35), a five-epoch warm-up followed by cosine learning-rate decay, mixed precision, gradient clipping, and EMA decay of 0.9993. Validation selected the base checkpoint and no TTA. The model contained 1,938,532 trainable parameters, required 1,679.02 s for training, and achieved 4.785 ms inference per sample. Segmentation images and masks were resized to 160 × 160. UNetBetter was trained for 90 epochs with AdamW and combined BCE, Dice, and focal loss weighted 0.40, 0.40, and 0.20, respectively, using batch size 16 and paired augmentation. The best validation-Dice checkpoint and threshold 0.95 were fixed for testing. The model contained 7,762,465 trainable parameters, required 3,274.48 s for training, and achieved 3.257 ms inference per sample.

## 4 Results

This section reports dataset organization, AI-assisted curation signals, technical integrity checks, exploratory morphology screening, image-quality findings, Grad-CAM fidelity, CUI distribution and sensitivity, active-curation performance, and FAIR readiness. Mask-area flags are interpreted as statistical review candidates rather than confirmed annotation errors.

### 4.1 Dataset organization, manifest generation, and metadata enrichment

Table 1 presents the structural outputs of the framework. Panel A displays the size and taxonomy of the corpus and demonstrates that the framework generated 6,000 sample-level records linked with 15,586 external manifest entries (21,586 unified records), combining both classification and segmentation perspectives. Panel B indicates that all classes are represented in both splits and across all three anatomical planes, with approximately balanced plane coverage (axial 1993, coronal 1981, sagittal 2026). Panel C shows that the enrichment layer recovers acquisition descriptors and consistency indicators with full or near-full coverage: every record has a recovered sequence, valid filename, and consistent split and label fields, and every segmentation pair has matched image and mask dimensions and filename correspondence. Panel D lists the enriched metadata categories carried per sample, showing that the manifest functions simultaneously as a dataset description and as a curation log. Field-level metadata completeness percentages for the raw dataset were not computed because the original BRISC 2025 release does not include a structured per-field metadata inventory. The FAIR readiness improvement scores reflect the sub-criteria rubric differences between the simulated pre-curation state and the post-curation enriched manifest.

**Table 1:** Dataset organization, curation manifest composition, acquisition descriptors, enrichment field coverage, and enriched metadata categories produced by NeuroFAIR Curator.

Panel/field	Value/coverage	Notes
A. Dataset summary		
Classification samples	6,000	5,000 train; 1,000 test
Segmentation image-mask pairs	4,793	Binary PNG masks
Classes	glioma; meningioma; pituitary; no_tumor	
Planes	axial; coronal; sagittal	
Dominant native size	512 × 512	4,881/6,000
External manifest entries	15,586	Source records
Framework sample records	6,000	Unique sample records
Unified linked records	21,586	Linked accounting total
B. Distribution		
Glioma	1,401	1,147 train; 254 test
Meningioma	1,635	1,329 train; 306 test
Pituitary	1,757	1,457 train; 300 test
No tumor	1,207	1,067 train; 140 test
Axial/coronal/sagittal	1,993/1,981/2,026	
C. Technical coverage		
Recovered sequence	6,000/6,000 (100%)	T1
Valid filename/split/label	6,000/6,000 (100%)	
Mask-image dimension match	4,793/4,793 (100%)	
Filename-mask correspondence	4,793/4,793 (100%)	

Figure 3 presents the dataset distribution across classes, splits, and planes recovered from the NeuroFAIR Curator manifest. Figure 3a shows the class distribution across glioma, meningioma, pituitary, and no tumor categories, indicating that all target classes are represented in the dataset. Figure 3b shows the split distribution, confirming the predefined division of 5,000 training samples and 1,000 testing samples. Figure 3c shows the anatomical plane distribution across axial, coronal, and sagittal views, demonstrating nearly balanced plane coverage.

**Figure 3:**
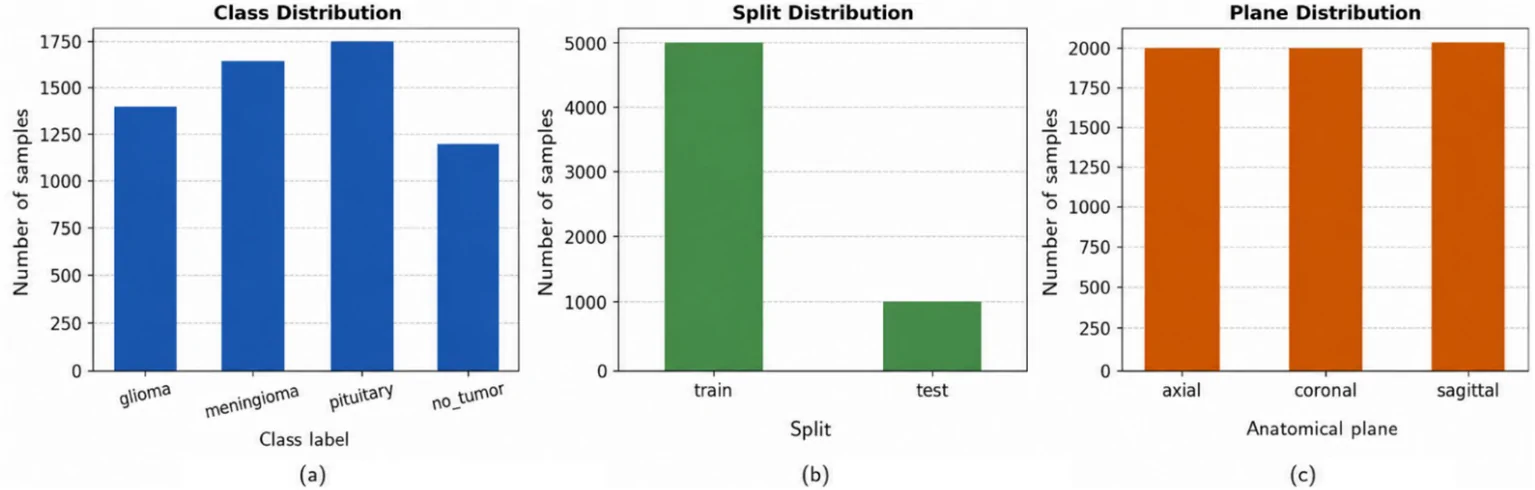
Dataset distribution: **(a)** tumor-class distribution across glioma, meningioma, pituitary, and no-tumor samples; **(b)** predefined training and test split distribution; and **(c)** anatomical-plane distribution across axial, coronal, and sagittal views.

### 4.2 AI-assisted curation model performance and per-class signal statistics

Table 2 summarizes classification and segmentation performance, per-class classification results, and computational overhead. The models are evaluated as curation instruments that generate confidence, uncertainty, and model-reference agreement signals rather than as clinical decision systems. The classifier achieved accuracy 0.9830, macro precision 0.9854, macro recall 0.9853, macro F1 0.9853, and macro AUC 0.9979. Per-class F1 ranged from 0.9736 for meningioma to 1.0000 for the no-tumor class. The segmenter achieved Dice 0.8230, IoU 0.7269, precision 0.8364, recall 0.8461, Hausdorff distance 7.842 pixels, HD95 4.111 pixels, and boundary quality 0.9964 at threshold 0.95. These values support computational mask screening but do not establish clinical annotation correctness.

**Table 2:** AI-assisted curation model performance, per-class classification results, and computational overhead.

Panel/item	Value	Notes
A. Classification		
Accuracy	0.9830	Test
Macro precision	0.9854	
Macro recall	0.9853	
Macro F1	0.9853	
Macro AUC	0.9979	
Best validation macro F1	0.9886	
Glioma P/R/F1	0.9842/0.9803/0.9822	*n* = 254
Meningioma P/R/F1	0.9833/0.9641/0.9736	*n* = 306
Pituitary P/R/F1	0.9739/0.9967/0.9852	*n* = 300
No tumor P/R/F1	1.0000/1.0000/1.0000	*n* = 140
B. Segmentation		
Dice	0.8230	Slice mean
IoU	0.7269	
Precision	0.8364	
Recall	0.8461	
Hausdorff distance	7.842 px	Pixels only
HD95	4.111 px	Pixels only
Boundary quality	0.9964	
Threshold	0.95	Validation-selected
Best validation Dice	0.8108	
C. Overhead		
ResCNNClassifier	1.939 M params	120 epochs; 1,679.02 s; 4.785 ms/sample; base checkpoint; no TTA
UNetBetter	7.762 M params	90 epochs; 3,274.48 s; 3.257 ms/sample

Figure 4 summarizes the performance of the two AI components used in NeuroFAIR Curator. Figure 4a shows strong label validation performance with limited off-diagonal misclassification across the four tumor classes. Figure 4b indicates stable convergence of the classification model, while Figure 4c shows progressive improvement of segmentation Dice with decreasing training and validation losses. Together, these results support the use of the classification and segmentation models as curation instruments for label validation and mask integrity assessment.

**Figure 4:**
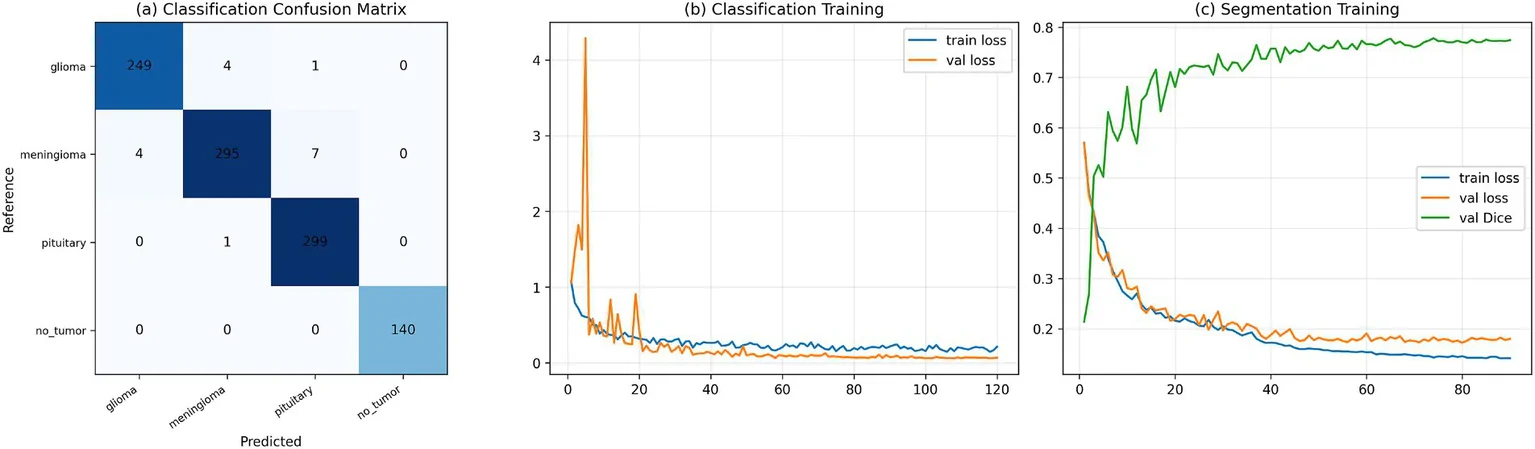
AI-assisted curation model performance. **(a)** Classification confusion matrix. **(b)** Classification training and validation loss. **(c)** Segmentation training loss, validation loss, and validation Dice curves.

Figure 5 presents four distinct representative segmentation examples from the held-out test set. For each case, the input MRI slice, corresponding ground-truth mask, predicted mask generated at the validation-selected threshold of τ = 0.95, and pixel-level error map are shown. The displayed Dice and IoU values quantify model–reference agreement for each individual sample. These examples illustrate segmentation-derived computational screening and boundary-level disagreement and are not interpreted as clinical validation of mask correctness.

**Figure 5:**
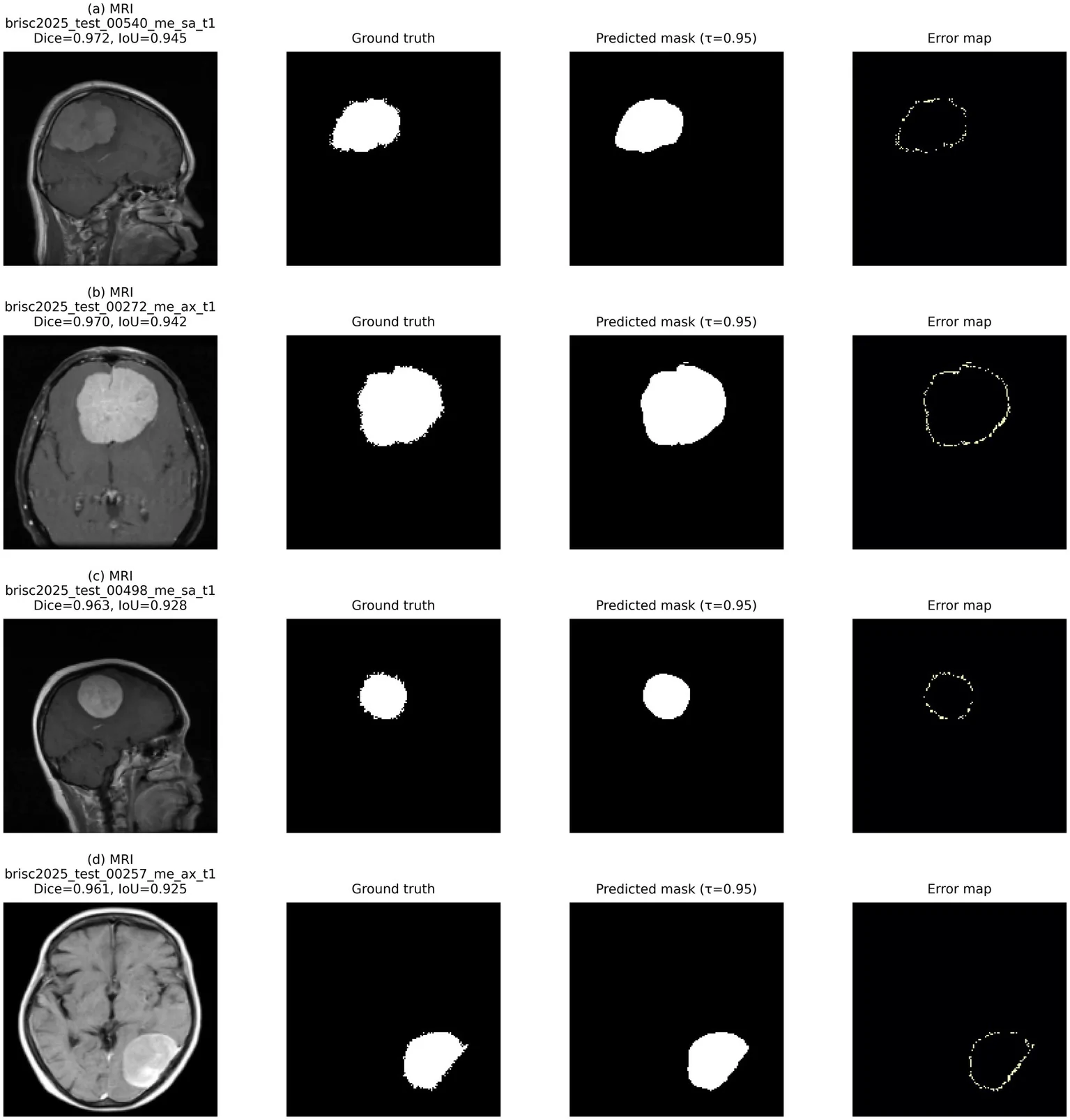
Representative qualitative examples of mask-integrity assessment, showing images, reference masks, and predicted masks. MRI images displayed in the figures originate from the BRISC 2025 dataset. Source: BRISC: Annotated Dataset for Brain Tumor Segmentation and Classification. Creative Commons Attribution 4.0 International (CC BY 4.0); the dataset is cited in the manuscript.

Figure 6 presents the distribution of tumor mask area ratios stratified by class and anatomical planes. Figure 6a shows the glioma masks, where sagittal and axial views display wider variation than coronal views. Figure 6b shows the meningioma masks, which have the broadest spread and the largest upper tail, especially in the axial plane, indicating greater variability in tumor mask size. Figure 6c shows the pituitary masks, which have comparatively smaller and more compact area ratios across all planes. Overall, the figure demonstrates that mask size distributions differ by tumor class and imaging plane, supporting the use of class-aware and plane-aware thresholds for detecting mask area outliers in the NeuroFAIR Curator pipeline.

**Figure 6:**
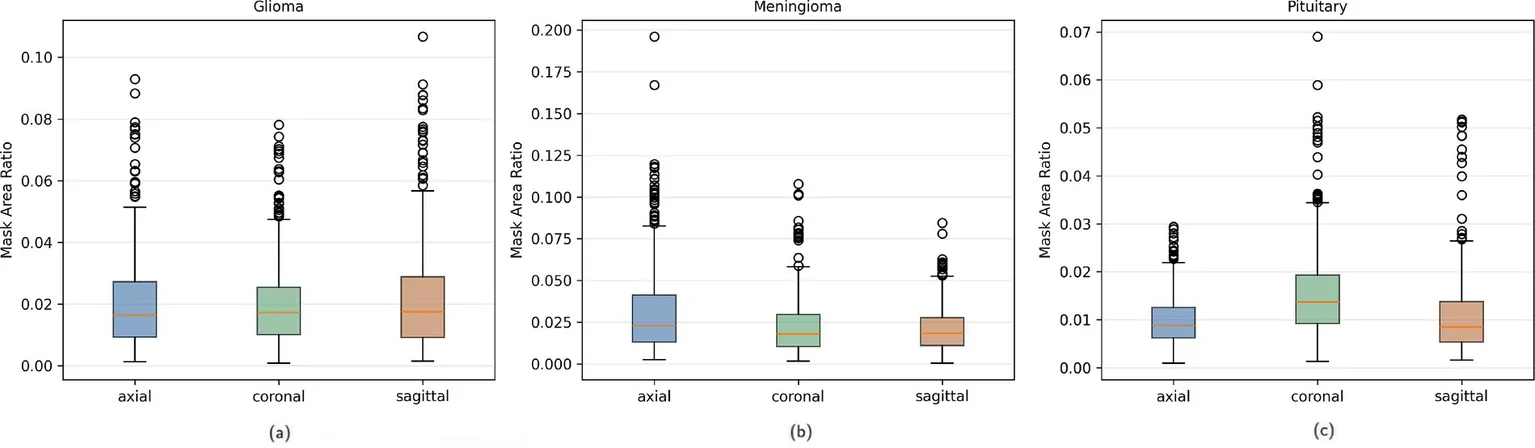
Distribution of supplied mask-area ratios by tumor class and anatomical plane: **(a)** glioma, **(b)** meningioma, and **(c)** pituitary tumors. The distributions support exploratory statistical morphology screening; flagged values are not interpreted as confirmed annotation errors.

Figure 7 presents Exploratory morphology characterization by tumor class and anatomical plane. Figure 7a Glioma mask-area ratios are shown for axial, coronal, and sagittal planes, with the sagittal group displaying a comparatively wider upper distribution and a larger number of high-area flags. Figure 7b Meningioma exhibits the broadest mask-area variability, particularly in the axial plane, where several markedly high values are observed, while the coronal and sagittal groups show more compact distributions with fewer extreme cases. Figure 7c Pituitary masks generally have smaller area ratios than glioma and meningioma, although the coronal plane shows a wider spread and more high-area flags than the axial and sagittal planes. In all subplots, colored cross markers represent samples identified using class- and plane-specific 1.5 × IQR limits.

**Figure 7:**
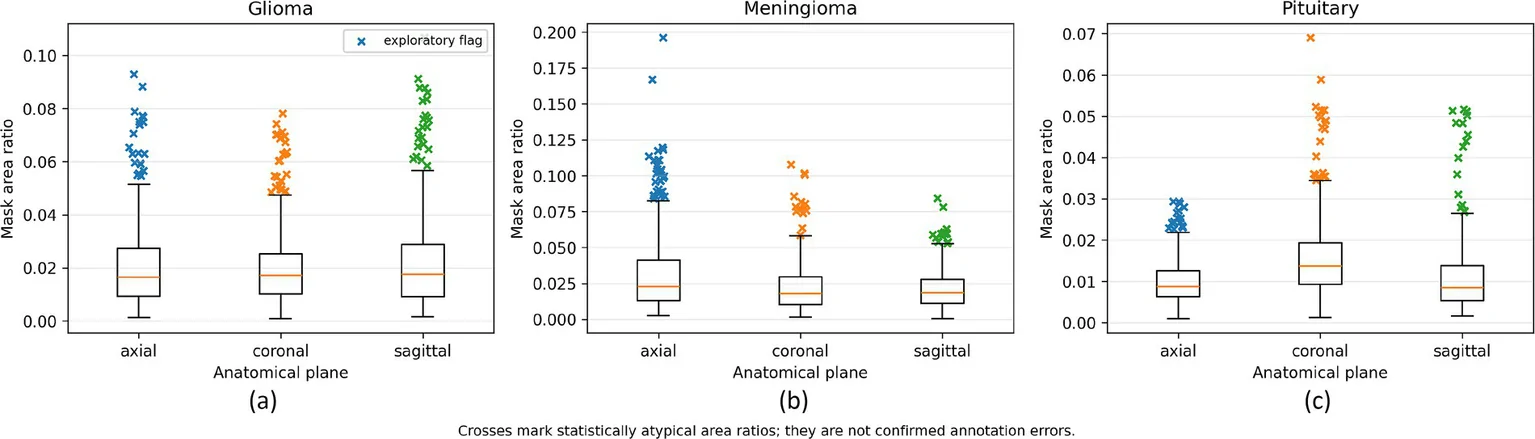
Exploratory morphology characterization by tumor class and anatomical plane: **(a)** glioma, **(b)** meningioma, and **(c)** pituitary mask-area ratios across axial, coronal, and sagittal planes. Cross markers indicate class- and plane-specific 1.5 × IQR statistical rarity and do not establish clinical annotation error.

### 4.3 Technical integrity, exploratory morphology, quality control, and CUI distribution

Table 3 shows that no missing tumor masks, empty tumor masks, dimension mismatches, filename inconsistencies, unknown planes, or corrupted samples were detected. Exploratory morphology screening identified 181 of 4,793 supplied masks (3.78%) outside class- and plane-specific 1.5 × IQR limits. These cases are statistically uncommon review candidates, not confirmed errors. Quality screening identified 51 low-contrast images, 60 blur outliers, 208 intensity outliers, and 180 Isolation Forest anomalies. Overall, 456 samples carried at least one flag and 184 carried multiple flags. The CUI assigned 5,001 samples (83.35%) to low curation priority and 999 samples (16.65%) to moderate curation priority.

**Table 3:** Technical annotation checks, exploratory morphology screening, image-quality findings, and CUI priority distribution.

Panel/check	Count	Percent/denominator
A. Objective technical checks		
Missing tumor mask	0	0.00% of 6,000
Large no-tumor mask	0	0.00%
Empty tumor mask	0	0.00%
Dimension mismatch	0	0.00%
Filename-label mismatch	0	0.00%
Filename-split mismatch	0	0.00%
Filename-mask mismatch	0	0.00%
Unknown plane	0	0.00%
B. Exploratory morphology		
Atypical mask-area flag	181	3.78% of 4,793
C. Quality and anomaly		
Low contrast	51	0.85%
Blur outlier	60	1.00%
Intensity outlier	208	3.47%
Isolation Forest anomaly	180	3.00%
Corrupted	0	0.00%
Any flag	456	7.60%
Multiple flags	184	3.07%
D. CUI priority		
Low priority	5,001	83.35%
Moderate priority	999	16.65%

Figure 8 summarizes the image quality control and anomaly analysis performed by the NeuroFAIR Curator. Figure 8a shows that most MRI samples have contrast values concentrated around the central range, while a small number of low-contrast or extreme-contrast cases appear in the distribution tails. Figure 8b shows that blur scores are highly skewed, with most samples concentrated at lower values and a small number of high-blur-score outliers. Figure 8c visualizes intensity-based quality variation by plotting image mean against image standard deviation, where samples with higher anomaly scores appear toward the sparse outer regions of the distribution. Figure 8d shows the anomaly score distribution, indicating that most images have low anomaly scores, while a smaller subset forms a high-score tail. Together, these subfigures support the image quality control module by identifying low contrast, blur, intensity outliers, and anomaly-flagged samples for downstream curation analysis.

**Figure 8:**
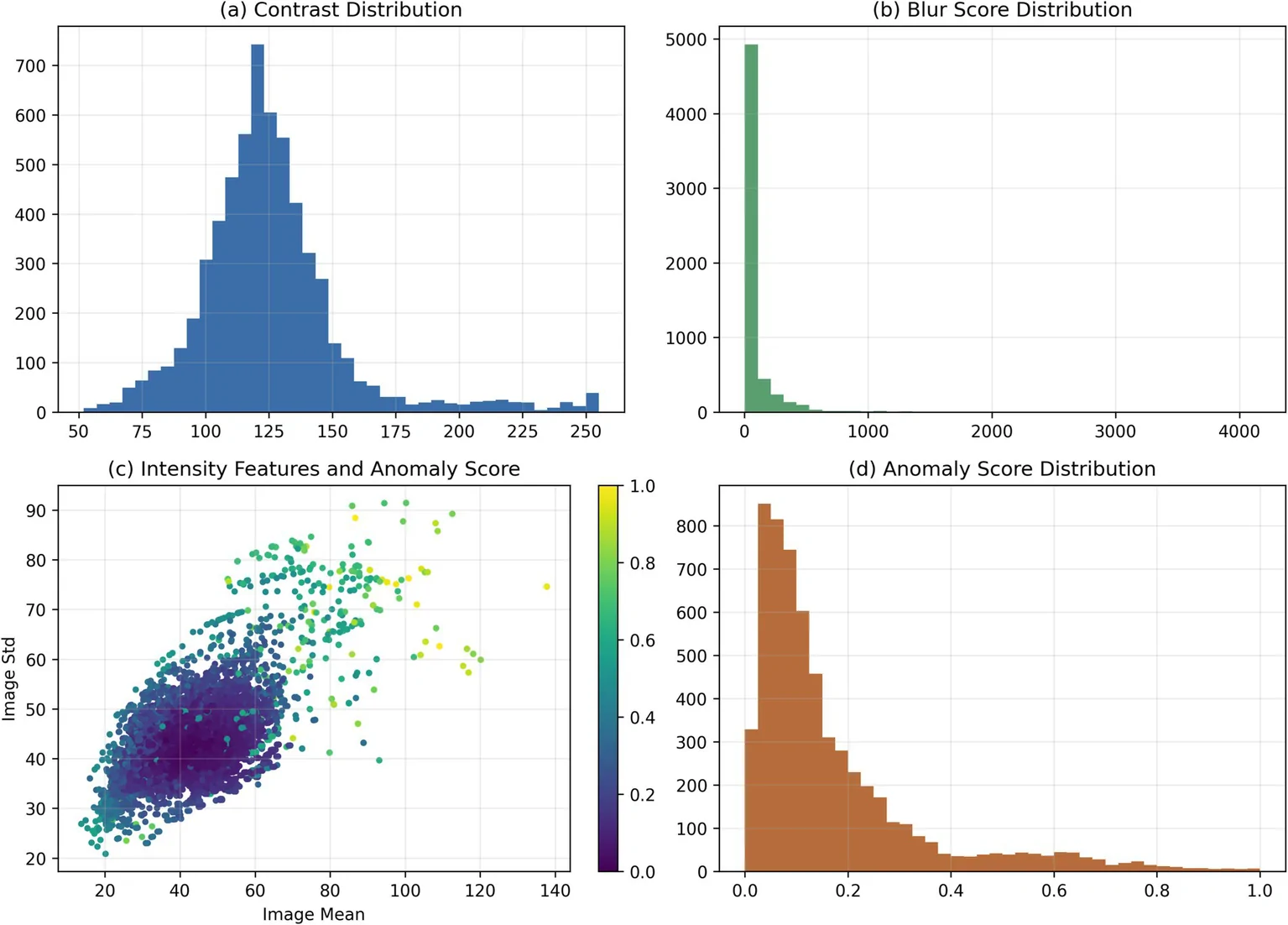
Image-quality and anomaly distributions: **(a)** Contrast distribution. **(b)** Blur-score distribution. **(c)** Image mean and standard deviation colored by anomaly score. **(d)** Image-quality anomaly-score distribution.

### 4.4 Explainability analysis (grad-CAM and DAUC)

Grad-CAM maps were generated from the final convolutional layer for the 20 highest-uncertainty test images. Fidelity was evaluated by progressively deleting the most salient pixels and integrating the target-class probability curve. Mean DAUC was 0.2996. Per-class means were 0.3145 ± 0.1028 for glioma (*n* = 6) and 0.2933 ± 0.2237 for meningioma (*n* = 14). Lower DAUC indicates a larger confidence reduction after salient-region deletion. These results quantify the faithfulness of the importance ordering but do not provide clinical localization validation as shown in Figure 9.

**Figure 9:**
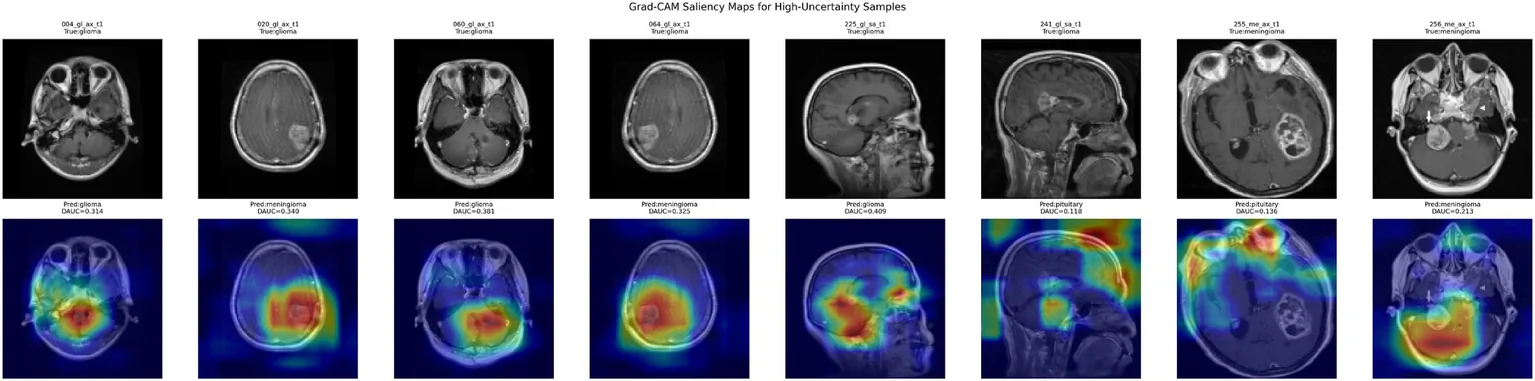
Grad-CAM maps for the 20 highest-uncertainty test images. Original MRI slices and saliency overlays are shown with DAUC values. Lower DAUC indicates a larger confidence reduction when salient pixels are deleted. MRI images displayed in the figures originate from the BRISC 2025 dataset. Source: BRISC: Annotated Dataset for Brain Tumor Segmentation and Classification. Creative Commons Attribution 4.0 International (CC BY 4.0); the dataset is cited in the manuscript.

### 4.5 CUI sensitivity, active curation, ablation, and FAIR readiness

Table 4 summarizes active curation, ablation, CUI sensitivity, and FAIR readiness. At review budgets of 1, 5, and 10%, the baseline CUI captured 49, 69, and 78 of 456 uniquely flagged samples, corresponding to 10.75, 15.13, and 17.11%. Most renormalized perturbations produced high rank stability. The largest ranking change occurred when segmentation uncertainty was reduced by 50% (Spearman *ρ* = 0.9925; mean top-k overlap = 75.28%; mean capture = 30.85%), while increasing the image-quality anomaly weight by 50% increased mean capture to 18.79% with a mean top-k overlap of 94.39%. Findability improved from 87.50 to 100.00 and Interoperability from 36.00 to 91.98; Accessibility and Reusability remained at 95.00 and 95.98.

**Table 4:** Active-curation capture, ablation analysis, CUI sensitivity summary, and FAIR readiness before and after curation.

Panel/item	Value 1	Value 2	Value 3
A. Active curation			
1% budget	60 reviewed	49 captured	10.75%; 99% reduction
2% budget	120 reviewed	63 captured	13.82%; 98% reduction
5% budget	300 reviewed	69 captured	15.13%; 95% reduction
10% budget	600 reviewed	78 captured	17.11%; 90% reduction
15% budget	900 reviewed	90 captured	19.74%; 85% reduction
20% budget	1,200 reviewed	99 captured	21.71%; 80% reduction

B. FAIR	Before	After	Improvement
Findability	87.50	100.00	+12.50
Accessibility	95.00	95.00	0.00
Interoperability	36.00	91.98	+55.98
Reusability	95.98	95.98	0.00

C. Ablation	Mean CUI		
Full	0.0677		
Without classification	0.0635		
Without segmentation	0.0281		
Without anomaly	0.0437		
Without metadata	0.0677		
Without FAIR	0.0677		

D. Sensitivity	Spearman ρ	Top-k overlap	Mean capture
Segmentation uncertainty −50%	0.9925	75.28%	30.85%
Image-quality anomaly +50%	0.9975	94.39%	18.79%

The sensitivity heatmap in Figure 10a shows that classification-uncertainty and mask-abnormality perturbations had limited effects on capture. Segmentation-uncertainty reduction and image-quality anomaly up-weighting produced the most visible changes. Figure 10b presents the final CUI priority distribution, and Figure 10c shows FAIR readiness before and after curation.

**Figure 10:**
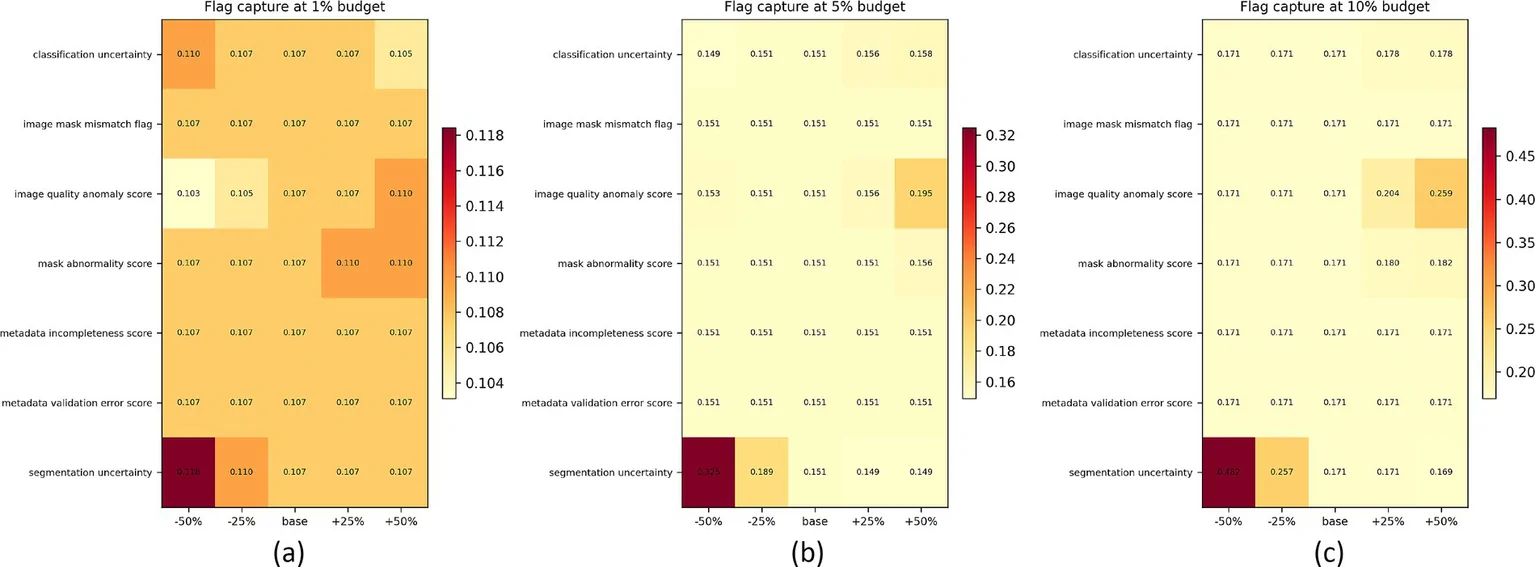
CUI sensitivity analysis following component-weight perturbation and renormalization: **(a)** flagged-sample capture at a 1% review budget, **(b)** capture at a 5% review budget, and **(c)** capture at a 10% review budget.

For context on the performance of NeuroFAIR Curator’s AI components used inside NeuroFAIR Curator, Table 5 compares the proposed framework with recent brain tumor MRI classification and segmentation studies. The comparison uses only numerical values reported in the literature and shows that most existing studies focus on classification or segmentation accuracy, whereas NeuroFAIR Curator additionally reports metadata enrichment, annotation integrity findings, CUI-based prioritization, and FAIR readiness improvement.

**Table 5:** Comparison with recent state-of-the-art brain tumor MRI studies.

Study	Dataset and input	Method/task	Reported performance	Parameters (M)	FLOPs (G)	Training requirement	Inference time	Curation/FAIR output
Díaz-Pernas et al. (2021)	CE-T1 MRI; 3,064 two-dimensional slices from 233 patients	Multiscale CNN for tumor classification and segmentation	Accuracy: 0.973	2.857	NR	80 epochs per fold; approximately 5 days	57.5 s/image	Not reported
Chen et al. (2019)	BraTS 2018; four-modality 3D MRI volumes	Dilated Multi-Fiber Network for lightweight 3D segmentation	Dice—ET: 0.8012; WT: 0.9062; TC: 0.8454	3.88	27.04	500 epochs	0.019 s/volume on GPU; 20.6 s/volume on CPU	Not reported
Isensee et al. (2020)	BraTS 2020; 3D multimodal MRI; 128 × 128 × 128 patches	Self-configuring 3D U-Net ensemble	Dice—ET: 0.8203; WT: 0.8895; TC: 0.8506	NR	NR	1,000 epochs per fold; final ensemble of 25 models	NR	Not reported
Wang et al. (2021)	BraTS 2019; four-modality 3D MRI; 128 × 128 × 128 patches	3D CNN–Transformer segmentation model	Dice—ET: 0.7893; WT: 0.9000; TC: 0.8194	32.99	333.00	8,000 epochs	NR	Not reported
Hatamizadeh et al. (2021)	BraTS 2021; 1,251 multimodal 3D MRI subjects; 128 × 128 × 128 patches	Hierarchical Swin Transformer for 3D segmentation	Dice—ET: 0.858; WT: 0.926; TC: 0.885	61.98	394.84	800 epochs; batch size 1 per GPU	NR	Not reported
Zhang et al. (2024)	BraTS 2019; 3D MRI volumes	CU-Net for brain-tumor segmentation	Overall Dice: 0.8241	NR	NR	Best validation checkpoint selected; duration NR	NR	Not reported
Nguyen et al. (2024)	BraTS 2019; four-modality 3D MRI; 128 × 128 × 128 patches	3D U-Net with Contextual Transformer	Dice—ET: 0.820; WT: 0.890; TC: 0.815; mean: 0.842	1.70	NR	100 epochs with three-fold cross-validation; 10.6 h/epoch as reported	NR	Not reported
Fateh et al. (2026)	BRISC; 6,000 two-dimensional CE-T1 MRI images and 4,793 image–mask pairs	EfficientNetB0 classification and SaberNet segmentation	Accuracy: 0.9920; weighted mIoU: 0.8060	NR	NR	NR	NR	Dataset and benchmark release; no CUI or FAIR-readiness assessment
NeuroFAIR Curator classifier	BRISC; 6,000 two-dimensional MRI images resized to 128 × 128	ResCNNClassifier for label validation	Accuracy: 0.9830;macro F1: 0.9853;macro AUC: 0.9979	1.939	NR	120 epochs; 1,679.02 s	4.785 ms/sample	Label-disagreement screening, uncertainty estimation, Grad-CAM and DAUC evaluation
NeuroFAIR Curator segmenter	BRISC; 4,793 two-dimensional image–mask pairs resized to 160 × 160	UNetBetter for mask-integrity screening	Dice: 0.8230;IoU: 0.7269;HD95: 4.111 px	7.762	NR	90 epochs; 3,274.48 s	3.257 ms/sample	Segmentation uncertainty, mask disagreement, boundary analysis and area-outlier detection
Complete NeuroFAIR Curator	BRISC unified metadata and curation resource	ResCNNClassifier + UNetBetter + QC + anomaly analysis + CUI + FAIR assessment	Not applicable as a single diagnostic metric	9.701	NR	Model-training subtotal: 5,092.29 s	Model-forward-pass subtotal: 4.972 ms/paired sample; complete pipeline runtime NR	Metadata enrichment, annotation auditing, image-quality analysis, Grad-CAM, CUI ranking, sensitivity analysis and FAIR-readiness evaluation

## 5 Discussion

The results support the use of classification and segmentation models as computational curation instruments rather than diagnostic endpoints. The unchanged ResCNNClassifier generated reliable label-confidence signals (accuracy 0.9830; macro F1 0.9853; macro AUC 0.9979), while the segmenter provided uncertainty and model-reference agreement signals (Dice 0.8230; boundary quality 0.9964). Exploratory morphology screening identified 181 masks whose area ratios were statistically uncommon for their tumor class and anatomical plane. Such cases may reflect tumor size, slice position, morphology, annotation convention, or genuine annotation problems. Because no clinical-reader assessment was performed, these explanations cannot be distinguished, and the flags must not be interpreted as confirmed mask errors.

Image-quality screening complements technical annotation checks by documenting low contrast, blur, intensity extremes, and multivariate anomalies. The CUI combines these heterogeneous signals into a transparent ranking for limited review budgets. Sensitivity analysis indicates that the ranking is stable under most reasonable weight perturbations, while segmentation uncertainty and image-quality anomaly contribute most strongly to ranking changes. The baseline weights should be interpreted as a transparent prespecified prior rather than clinically calibrated coefficients.

The FAIR analysis indicates that the principal improvements occur in Interoperability (+55.98) and Findability (+12.50). Accessibility and Reusability did not change because the declared pre-curation rubric already assigned high scores to those dimensions. This pattern reflects the selected 18-sub-criterion instrument and BRISC 2025 and should not be presented as universal. The enriched manifest nevertheless adds harmonized descriptors, quality indicators, curation signals, and provenance fields that support reproducible reuse.

The selected threshold of 0.95 is specific to the BRISC 2025 validation distribution and is not a universal NeuroFAIR Curator parameter. Differences in scanner hardware, contrast enhancement, acquisition protocol, spatial resolution, preprocessing, intensity normalization, and artifact prevalence can alter calibration. For transfer to a new dataset, threshold tuning should be repeated on a representative validation subset and fixed before test evaluation. Limitations include evaluation on one two-dimensional JPEG/PNG corpus, absence of radiologist assessment, unavailable reliable pixel spacing and volumetric registration, prespecified rather than clinically calibrated CUI weights, and dependence of FAIR results on the selected rubric.

## 6 Conclusion

NeuroFAIR Curator provides an AI-assisted and reproducible framework for transforming public brain tumor MRI data into a metadata-enriched, technically checked, quality-screened, review-prioritized, and FAIR-aligned neuroinformatics resource. Applied to BRISC 2025, the framework generated 6,000 sample-level curation records linked with 15,586 external manifest entries and processed 4,793 image–mask pairs. The unchanged ResCNNClassifier achieved an accuracy of 0.9830, macro-F1 of 0.9853, and macro AUC of 0.9979, while the segmentation model achieved a Dice score of 0.8230 and an IoU of 0.7269 using the validation-selected operating threshold of 0.95. Class- and plane-specific exploratory morphology screening identified 181 of 4,793 masks (3.78%) with statistically uncommon area ratios. These cases were retained as candidates for prioritized review and were not interpreted as confirmed annotation errors.

The main contribution of NeuroFAIR Curator is the integration of metadata enrichment, classification-derived label screening, segmentation-derived uncertainty analysis, objective technical checks, image-quality control, exploratory mask morphology characterization, transparent CUI computation, sensitivity analysis, active-curation prioritization, and FAIR readiness evaluation within a single auditable workflow. The FAIR assessment showed that the principal gains occurred in Interoperability (+55.98) and Findability (+12.50), reflecting the addition of structured descriptors, quality indicators, provenance information, and machine-actionable curation signals. NeuroFAIR Curator should therefore be understood as a computational data-stewardship and review-prioritization framework rather than a clinical validation system. It does not replace radiologist assessment or establish the anatomical correctness of supplied masks. Future work should evaluate the framework using blinded expert review, multicenter DICOM or NIfTI datasets, three-dimensional annotations, and external calibration across different scanners, acquisition protocols, contrast conditions, and artifact profiles.

## Data Availability

The original contributions presented in the study are included in the article/supplementary material, further inquiries can be directed to the corresponding author.
